# Understanding Current Density in Molecules Using Molecular Orbitals

**DOI:** 10.1021/acs.jpca.3c04631

**Published:** 2023-10-19

**Authors:** William Bro-Jørgensen, Gemma C. Solomon

**Affiliations:** † Department of Chemistry and Nano-Science Center, University of Copenhagen, Universitetsparken 5, DK-2100 Copenhagen, Denmark; ‡ NNF Quantum Computing Programme, Niels Bohr Institute, University of Copenhagen, 2100 Copenhagen, Denmark

## Abstract

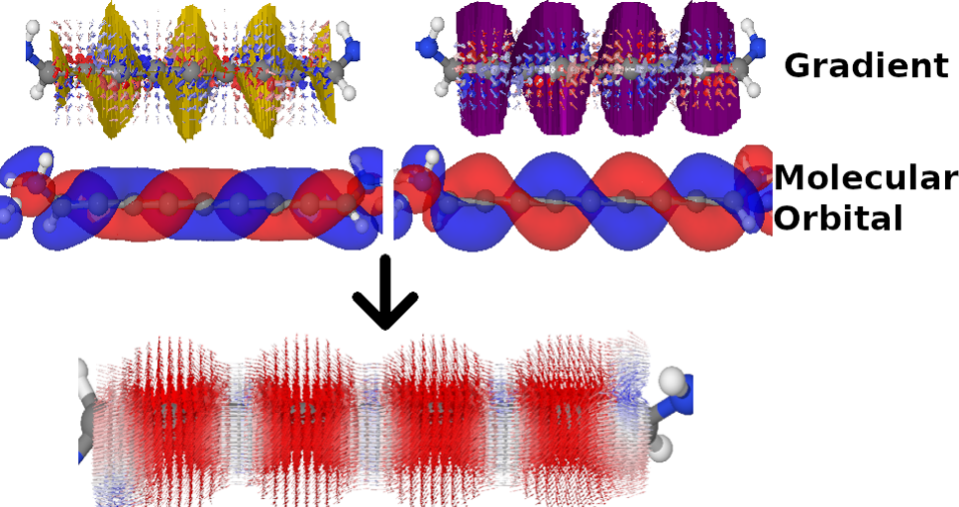

While the use of molecular orbitals (MOs) and their isosurfaces to explain physical phenomena in chemical systems is a time-honored tool, we show that the nodes are an equally important component for understanding the current density through single-molecule junctions. We investigate three different model systems consisting of an alkane, alkene, and even [*n*]cumulene and show that we can explain the form of the current density using the MOs of the molecule. Essentially, the MOs define the region in which current can flow and their gradients define the direction in which current flows within that region. We also show that it is possible to simplify the current density for improved understanding by either partitioning the current density into more chemically intuitive parts, such as σ- and π-systems, or by filtering out MOs with negligible contributions to the overall current density. Our work highlights that it is possible to infer a non-equilibrium property (current density) given only equilibrium properties (MOs and their gradients), and this, in turn, grants deeper insight into coherent electron transport.

## Introduction

The use of molecular orbitals (MOs) to qualitatively predict and explain chemical reactivity and physical phenomena is ubiquitous. Despite being a mathematical construct rather than a physical observable, MOs have nonetheless been successfully used to explain and predict physical effects.^1−6^

One interesting quantity that is calculated as an interaction between all MOs is the current density, and a variety of methods have been developed to calculate it through molecular junctions.^7−18^ It is a tool that provides spatial information about how the current flows through a molecular junction induced by a bias, meaning we can probe how the electronic properties of a molecular system change as a function of position in the molecule. A better understanding of the current density will also enable us to make more accurate predictions about the magnetic fields that will inevitably be created.^11,13,16^

**Figure 1:**
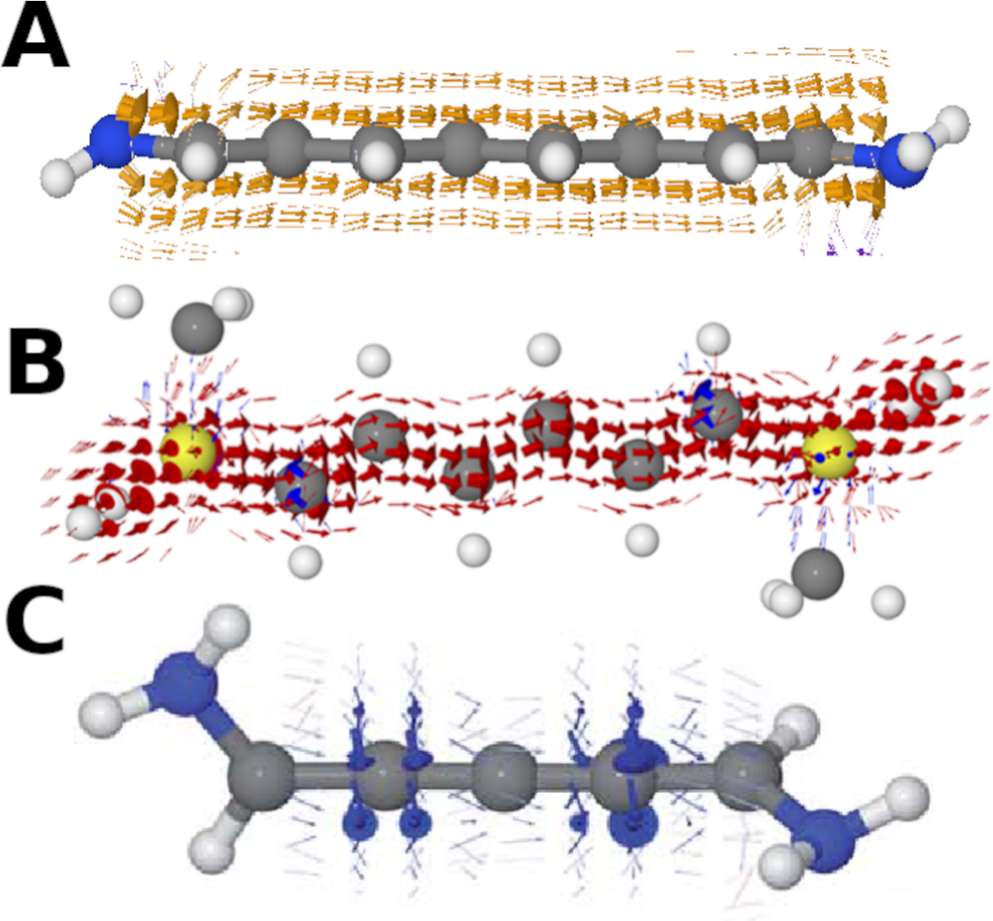
Illustration of three systems with nonintuitive current density. (A) Current density of 1,8-diaminooctatetraene (B) current density of 1,4-bis(methylthio)hexane. Reproduced with permission from ref (19). Copyright 2019 American Chemical Society. (C) Current density of 1,5-diamino-[4]cumulene. Reproduced with permission from ref (20) Copyright 2019 Royal Society of Chemistry. Note that in (B), no bonds are included (as per the original publication) so that the current density is more clearly visible.

In prior work, it has been noted that the current density through molecules sometimes seems to be at odds with our chemical intuition. In graphene and unsaturated polyenes,^11,16^ the current density has a nodal plane that spans the same plane as the molecule, where the current density goes to ≈0. We show an example of this in Figure 1A. At first, this seems to make intuitive sense as it is the same nodal plane that exists in all the π-MOs; however, no such clear relationship exists between MOs and current density for other systems, raising the question of why this is the case for these systems?

In alkanes,^19^ the current density seems to take the shortest path through the molecules, ignoring the zigzag path outlined by the σ-bonds. This pattern is preserved when moving to silanes and germanes and seems to suggest that bonds are not a good predictor for where the current will flow. We show an example of this current density in butane in Figure 1B.

The last system we investigate is even [*n*]cumulenes, where *n* and “even” refer to the number of cumulated double bonds. Formally, these molecules have D_2d_-symmetry, but when the symmetry is reduced to C_2_ (or D_2_) by substitution at the terminal carbon atoms, the orbitals become helical.^20−27^ Naturally, this might lead one to expect that the resulting current density will also be helical, but instead circular currents arise. We show an example of this current density in Figure 1C. Furthermore, these systems seem to contradict the conclusions drawn for conjugated molecules, such as polyenes and graphene, as we no longer see nodal planes in the current density—neither helical nor planar.

While we focus on even [*n*]cumulenes in this paper, the helical MOs are also seen in appropriately substituted polyynes,^28,29^ odd [*n*]cumulenes,^29^ metallacumulenes or -polyynes,^30−33^ spiro-compounds,^34^ and cyclo-allenes.^35,36^ We choose to focus on the even [*n*]cumulenes as these have helical MOs in their ground state, and long molecules result in long helical MOs, which aids visualization.

Here, we probe the components of current density and show that we can gain a deeper understanding of how current generally flows through molecules from the MOs alone. This contrasts with prior studies that primarily assessed the current density in particular molecules.^19,20^ First, we outline the formal definition of current density with an emphasis on making the equations simpler to use and easier to illustrate. Second, we show that the current density consists of simple building blocks that can be used to partition the current density into chemically intuitive subdivisions, such as σ- and π-systems. Finally, we explain the current density in the three model systems outlined above where neither interatomic local currents^37^ nor transmission eigenchannels^38^ can satisfactorily explain the current density.

## Methods

All molecular structures were optimized using density functional theory (DFT) using the Perdew–Burke–Ernzerhof^39,40^ (PBE) exchange–correlation functional as implemented in Gaussian16.^41^ The Pople basis set 6-311G(d,p) of triple-ζ quality augmented with polarization functions was employed.^42^

All current density calculations have been carried out using DFT as implemented in the Atomic Simulation Environment (ASE) and GPAW.^43−45^ The PBE exchange–correlation functional and a double-ζ plus polarization basis set were used for all atoms except hydrogens, where a single-ζ basis set was used. We set the Fermi energy to the midpoint between the energies of the highest occupied molecular orbital (HOMO) and the lowest unoccupied molecular orbital (LUMO). Therefore, unless otherwise noted, all current density results are calculated at (*E*_HOMO_ + *E*_LUMO_)0.5 = *E*_F_.

All structures and input files are available at https://iochembd.chem.ku.dk/browse/handle/100/52.

Due to computational constraints, the low-bias current density through our single-molecule junctions is calculated using s-band electrodes in the form of dihydrogen. The code is available at https://github.com/chem-william/calc_current.

To calculate electron transport through a molecule, we have to construct a suitable Hamiltonian. For this, we consider three regions: the left electrode, L; the right electrode, R; and the central region (the molecule), C. We write the Hamiltonian in an orbital basis as
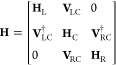
1assuming no direct interaction between the left and right electrodes. Here, **V**_LC/RC_ are the coupling matrices between the central region and electrode, **H**_L/R_ are the Hamiltonians for the left and right electrodes, respectively, and **H**_C_ is the Hamiltonian for the central region. In this case, **H**_C_ is the Hamiltonian for the molecule alone.

The general equation for calculating the current density, **J**, from a wavefunction, Ψ, is given by^46^
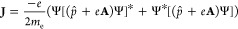
2where *p̂* ≡ −*i*ℏ∇ is the momentum operator, ℏ is the reduced Planck constant, *e* is the electronic charge, **A** is the vector potential of an external electromagnetic field, *m*_e_ is the mass of the electron, and Ψ is the wave function of the single electron of interest. This can be rewritten as
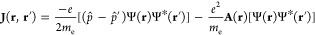
3where the gradient operators, ∇ and ∇′, operate on **r** and **r**′, respectively. In the following, we assume that **A**(**r**) = 0, and thus the second term of eq 3 disappears. Furthermore, we assume steady-state conditions and use the following relation as described by Datta^7^
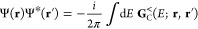
4where ***G***_C_^<^(*E*;**r**,**r**′) is the lesser Green’s function. Inserting this equation into eq 3, we arrive at eq 5
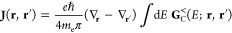
5where we have expanded the momentum operators. We transform the lesser Green’s function to a real-space representation by an orthonormal and complete atomic orbital (AO) basis {ψ_*i*_(**r**)}
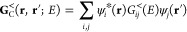
6Here, *G*_*ij*_^<^(*E*) are the individual elements of **G**_C_^<^(*E*;**r**,**r**^′^). We drop the “C”-subscript when referring to individual elements for notational clarity. Inserting eq 6 into eq 5, we obtain
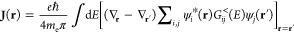
7which is rearranged to give eq 8, the current density per spin
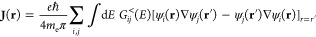
8

We have dropped the complex conjugate of ψ(**r**) as the AO basis has no imaginary component.

Under steady-state conditions, the lesser Green’s function is defined as
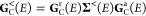
9where **G**^r^ and **G**^a^ are the retarded and advanced Green’s functions, respectively, and **Σ**^<^ is the lesser self-energy. The retarded and advanced Green’s function are defined as
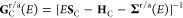
10where **S**_C_ is the overlap matrix of the central region and **Σ**^r/a^ is the retarded/advanced self-energy. The **Σ**^r/a^ connects the electrodes and the molecule. It is defined in terms of the coupling between the left and right electrodes and the central region
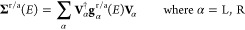
11where the non-interacting Green’s functions of the electrodes are
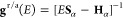
12

The final component of eq 9 is the lesser self-energy and is defined as
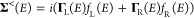
13where the spectral densities, Γ_L/R_(*E*), are given by
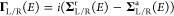
14and the Fermi functions by
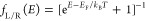
15Here, *E*_F_ is the Fermi energy, *k*_B_ is the Boltzmann constant, and *T* is the temperature. In the limit of zero temperature, we can model the Fermi functions as step functions
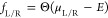
16

Furthermore, by assuming a small bias, , and that we stay at energies between the chemical potentials of the two leads, i.e., μ_R_ ≤ *E* ≤ μ_L_, we have that *f*_L_ = 1 and *f*_R_ = 0. This reduces **Σ**^<^ to
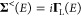
17and thus eq 9 becomes
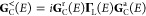
18

In the following, we will show that eq 8 can be rewritten to allow for easier interpretation. For **G**_C_^<^(*E*), the following relation holds
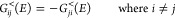
19

If we calculate two terms from the summation from eq 8
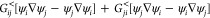
20where the constants  and the energy dependence have been omitted for notational simplicity.

By expanding the parentheses
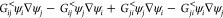
21and using the relation from eq 19, we get
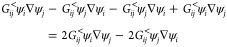
22and then again using eq 19, we reduce eq 22 to
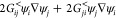
23

As eq 23 is two terms from the summation of eq 8, we can rewrite eq 8 as
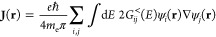
24

The current density can be calculated in either an AO or an MO basis. The transformation between bases is explained in the Supporting Information.

Although theoretically current density is conserved,^47^ in practice it is less clear. Our group previously investigated if, for example, increasing the size of the basis set could address this issue but found no substantial improvement.^19^ Nonetheless, the deviations were typically minimal and did not significantly alter the shape of the current density. Within the Supporting Information, we present both the total and integrated current from the current density, plotted against the *z*-coordinate (transport direction), for all studied molecules, underscoring that the integrated current density closely aligns with the total current density.

Equation 24 shows that the current density is a sum over orbital–gradient pairs, ψ_*i*_(**r**)∇ψ_*j*_(**r**), with *G*_*ij*_^<^(*E*) scaling how much each pair contributes to the overall current density. Therefore, it is straightforward to partition the current density into contributions that are closer to our chemical intuition. For example, we can choose to only look at the contributions from a subset of orbitals, for example: AOs of the same type, such as p-orbitals; individual MOs, such as the HOMO; or the full σ-system. We will use these types of subdivisions to explain how the current density manifests in the three model systems.

Another way to simplify the current density picture is to filter according to the value of **G**_*C*_^<^(*E*). Formally, we define the following quantity:

For *x* ∈ [0, 1]
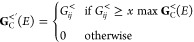
25

At *E*_F_, this is a poor approximation, as almost all MOs contribute to the current density. Rather, it becomes a useful tool when we calculate the current density at energies close to resonance features in the transmission, such as at the energy of the HOMO or the LUMO. At a resonance, only a few MOs have a significant contribution to the current density. This fact allows us to distinguish between emergent features that arise from the combination of many MOs and features that are intrinsic to a given MO-gradient pair.

Depending on which molecule we look at, it will prove instructive to analyze the current density in either a Cartesian or cylindrical coordinate system. This conversion is trivial and is included in the Supporting Information for reference. Each arrow is colored by either its normalized *z*- or θ-component according to the following set of equations
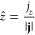
26
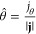
27Here, the hat denotes a scaled *z*- or θ-component, *j*_z/θ_ is the *z*- or θ-component at a given point, and **j** is the full vector at that point.

In addition, to convey a sense of where the current flows, all arrows are scaled according to their *z*-component and multiplied by a factor relative to the largest arrow
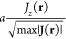
28Here, *a* is a scaling factor that is chosen to be  unless otherwise mentioned.

## Results and Discussion

**Figure 2:**
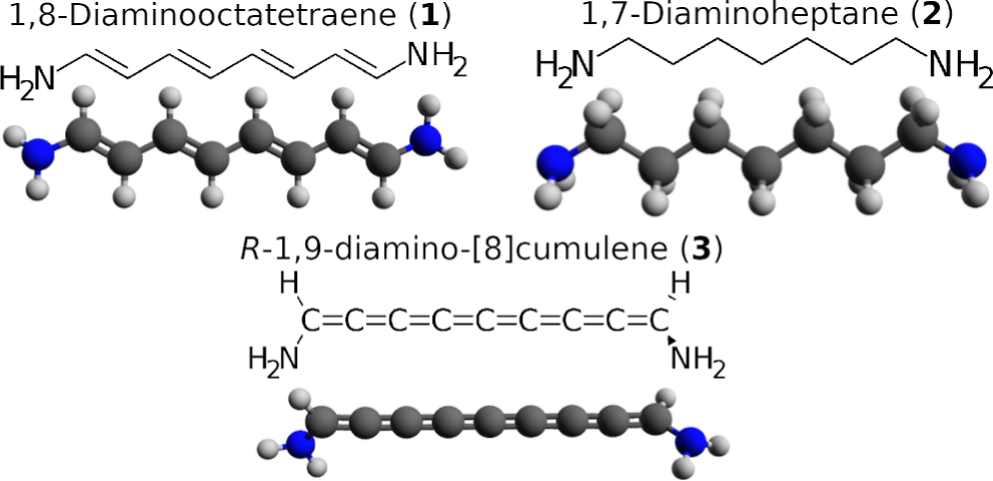
Name, chemical structure, and optimized molecular geometries of 1,8-diaminooctatetraene (**1**), 1,7-diaminoheptane (**2**), and R-1,9-diamino-[8]cumulene (**3**).

We will go through the three model systems shown in Figure 2:

1. Conjugated system in the form of a polyene (**1**), where the current runs primarily through the π-system. As all carbons are sp^2^-hybridized, it is similar to the graphene systems previously investigated.^11,17,48^
2. Alkane, in the form of heptane (**2**), where the current density curves away from the bonds instead of following them.
3. An even α, ω-disubstituted [*n*]cumulene (**3**) where the current density flows circularly around the carbon atoms. The length of **3** is challenging to reach synthetically,^49−53^ but it is chosen to emphasize the circular current density.

**Figure 3:**
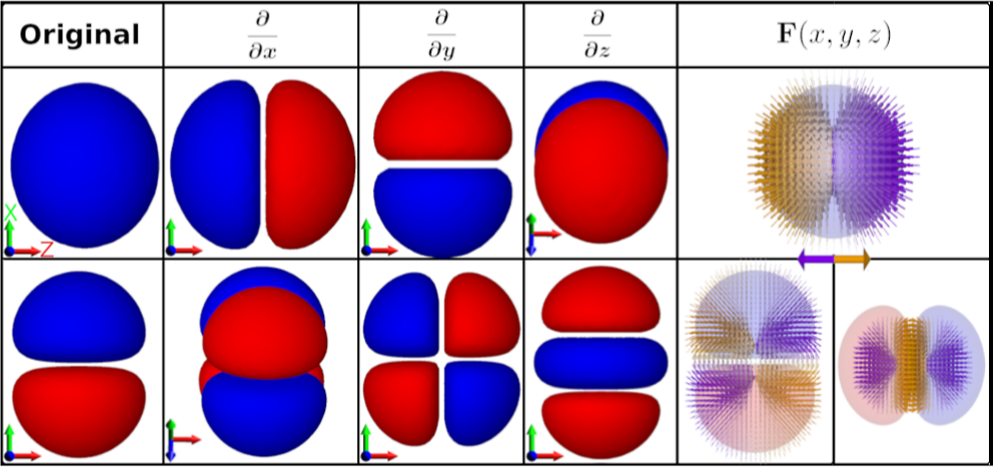
Comparative visualization of 1s- and 1p_*x*_-orbitals, their respective gradients in the *x*-, *y*-, and *z*-directions, and the resultant vector fields. (Upper) s-orbital and (lower) p-orbital. Only the gradient of a 1p_*x*_-orbital is shown, as the gradient of 1p_*x*_, 1p_*y*_, and 1p_*z*_ is fundamentally the same. The red and blue colors correspond to the sign of the isovalue. The vector field, **F**(*x*, *y*, *z*), is calculated for both an s, p_*x*_-, and p_*z*_-orbital to illustrate how **F**(*x*, *y*, *z*) changes with different orbitals.  of the s-orbital and  of the p_*x*_-orbital have been slightly tilted along the *z*-axis, so all lobes can be seen. The vector field **F**(*x*, *y*, *z*) is color-coded by the *z*-component, with left-pointing arrows in purple and right-pointing arrows in orange.

Before we consider our three molecular systems, it is important to first understand the basic AOs and their gradients that serve as foundational elements of the current density. Therefore, we illustrate the orbitals and gradients of eq 24 using a 1s- and 1p-orbital in Figure 3. These two atomic orbitals constitute the simplest building blocks of the more complex MOs that we will look at later. The *x*-, *y*-, and *z*-components of the gradient of a 1s-orbital are displayed in the top row, while the analogous components of a 1p_*x*_-orbital are depicted in the row beneath. The last column shows the resulting vector field, **F**(*x*, *y*, *z*), which is calculated as **F**(*x*, *y*, *z*) = ∇ψ, where ψ is either an s-, a p_*x*_-, or a p_*z*_-orbital. Of note is the substantial gradients across the nodal planes [see **F**(*x*, *y*, *z*) of the p_*x*_- or p_*z*_-orbital], which, as we will demonstrate later, play a key role in the path of the current density through molecules.

A curiosity to note is that taking the gradient of an AO seems to result in an AO with an orbital quantum number, *l*, that is one greater. For example,  of the s-orbital qualitatively matches a p_*x*_-orbital, while  of the p-orbital resembles d_*z*^2^_.

Note that all diagonal terms of eq 24 are equal to 0, irrespective of whether we calculate **J**(**r**) in an AO- or MO-basis. It is perhaps most clearly seen from eq 8, where  when *i* = *j*. Thus, viewed from an AO perspective, it is rarely the case that ∇ψ is multiplied with an AO of the same type. For example, the **F**(*x*, *y*, *z*) from an s-orbital might gain a node by being multiplied with a p-orbital, or the **F**(*x*, *y*, *z*) of a p-orbital might maintain its node-spanning vector field because it is multiplied with an s-orbital. The same type of interactions are also present if we change to an MO basis, though more complex.

**Figure 4:**
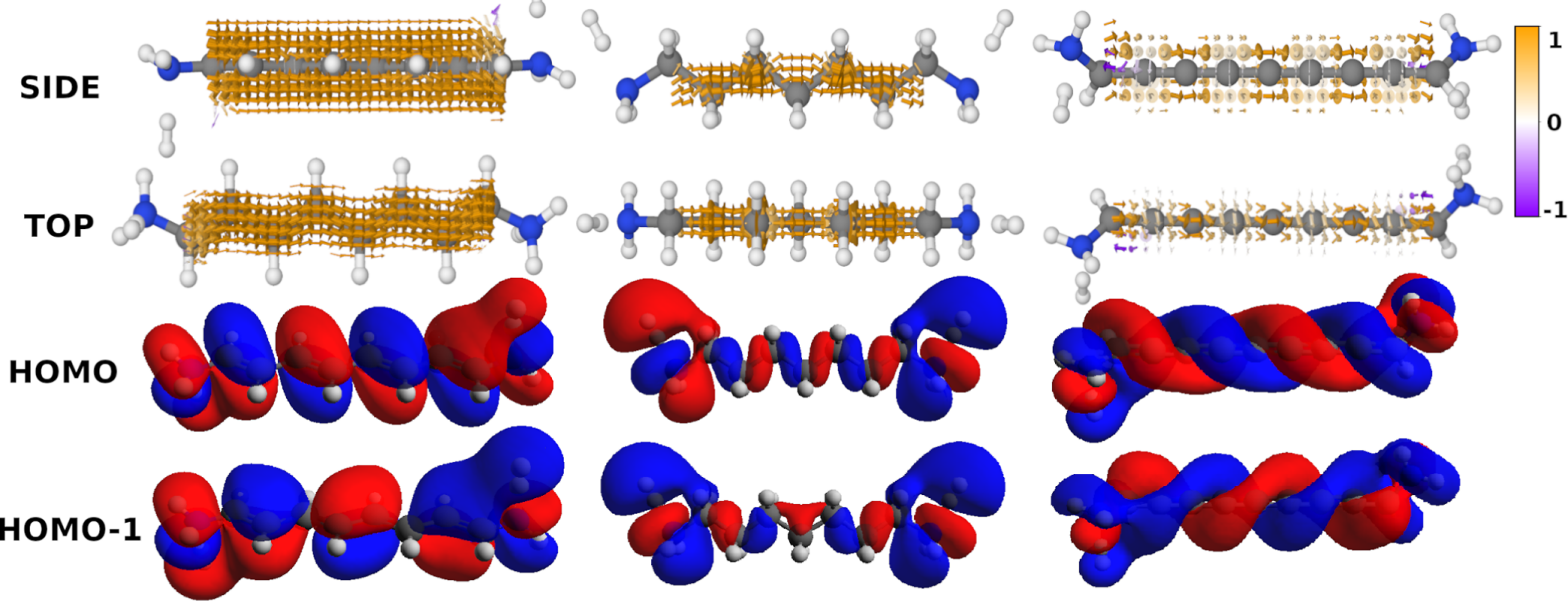
Current density calculated at *E*_F_ and the two HOMOs for all three model systems. (Left column) **1**, (middle column) **2**, and (right column) **3**. The vector fields are colored by the normalized *z*-component. Arrows smaller than 5% of the largest arrow have been omitted. Note, for **3**, the current density runs circularly around every other carbon atom. Isosurface: 0.01 au.

In Figure 4, we show the full current density of all three model systems and their two HOMOs. If we only consider their MOs, it is difficult to predict the qualitative shape of the current density in the three systems. In **1**, the nodal plane that spans the plane of the molecule is present in the current density, but not the nodal planes between the individual segments of the MOs; in **2**, the current density curves away from the bonds even though the MOs have their highest isovalues at the bonds; in **3**, the current density flows circularly around each atom while the MOs have a helical shape. Even though a helical nodal plane is seen in both of the HOMOs, no nodal plane can be seen in the current density. This seems to be in direct disagreement with what we see for **1**, where the nodal plane of the HOMO and HOMO – 1 shows up in the current density.

### Why Do We Sometimes See Nodes in the Current Density?

**Figure 5:**
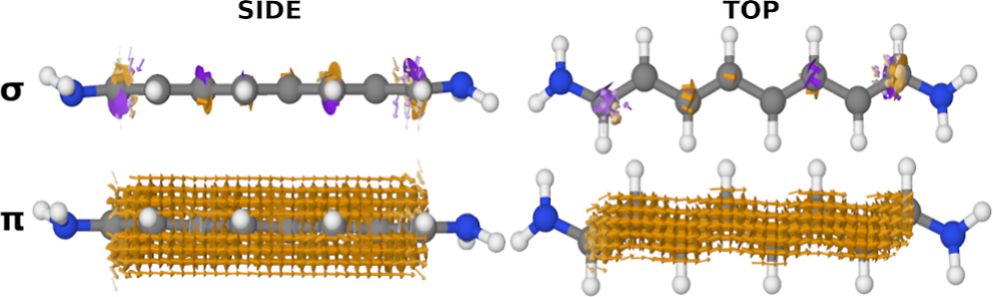
Current density of **1** calculated at the Fermi energy and partitioned into σ- and π-systems. The vector field is colored by the normalized *z*-component. Arrows smaller than 5% of the largest arrow of the total current density have been omitted.

In Figure 5, we show the current density of **1** partitioned into contributions from the σ- and π-system. If the σ- and π-contribution were added together, we get exactly the current density shown in the left column of Figure 4. Note that, while the contribution from the σ-system seems substantial as per the size and amount of the arrows, this is due to how the arrows are scaled, filtered, and plotted. The contributions from the σ- and π-systems are plotted separately, meaning that the arrows of the σ-system are scaled and filtered according to the largest arrow of the σ-system. Likewise for the π-system.

The contribution from the σ-system is small but not absent. This highlights one of the advantages of our method, as the current density from the σ-system is no longer masked by the electronic transmission through the π-system. As we know from systems where there is destructive quantum interference in the π-system, such as meta-substituted benzene^1,13,54−57^ or cross-conjugated polyenes,^55,58−60^ there can still be notable electronic transmission through the σ-system.^58^ This partitioning can help clarify how the current flows through molecules, regardless of which MOs dominate the overall picture.

The fact that the π-system dominates the current density matches our intuition from Hückel theory and tight-binding models, where the description of conjugated systems can be reduced to the π-system formed with p_*z*_-orbitals while qualitatively still describing many of the molecular properties.^61^

In the discussion of **1**, we will now ignore the σ-system and only focus on the contribution from the π-system to the current density. We showed in Figure 3 [see the gradients of the MOs, **F**(*x*, *y*, *z*)] that the gradient across a nodal plane is substantial. However, considering that ∇ψ is not necessarily multiplied with a ψ exhibiting a node in the same place, we ask the following question: Why is it that only the nodal plane in the plane of **1** is visible in the current density and not the other nodes of the MOs? For instance, why are the nodes in the HOMO and the HOMO – 1 between the bonds not apparent?

We first answer this question assuming an AO basis where every ψ is a p_*z*_-orbital. Here, every ∇ψ is multiplied with a ψ that bears a nodal plane in the plane of the molecule. As a consequence, any contribution to the current density will be exactly zero in the plane of the molecule. This principle is broadly applicable, in particular for systems where their current density is predominantly governed by their π-system, such as graphene. For the current density across bonds, ∇ψ is multiplied with a ψ that has a nonzero magnitude. The absence of a nodal plane between different AOs across the bonds prevents any suppression of the current density. If we switch our perspective to an MO basis, the story remains similar: each ψ will still have a guaranteed nodal plane in the plane of the molecule. This implies that, regardless of how strong the contribution across the nodal plane from ∇ψ might be, that contribution will be suppressed. Yet, upon turning our attention to the HOMO and HOMO – 1 (see the bottom row of Figure 4), we encounter a shift in the story. We observe that the nodes positioned between bonds vary in location among different orbital–gradient pairs. This lack of consistency means there is no uniform suppression of the current density, leading to the absence of nodal planes between bonds in the resulting current density.

In the previous discussion, we ignored the contribution to the current density from polarization functions. We show in Figure S9 that a small part of the current density through the π-system is explained by polarization functions that can form π-bonds. In our calculations, the polarization functions consist of d-functions, which means that, by symmetry, only d_*xz*_ can form π-bonds: with itself (d_*xz*_ – d_*xz*_) or with p_*z*_ (p_*z*_ – d_*xz*_). Both of these bonds also have a node in the plane of the molecule, thus the conclusion is the same. See Figure S10 for a plot of a d_*xz*_-orbital.

### Why Might the Shape of the Current Not Follow the Bonds?

In Figure 7, we show the current density of **2** calculated at two different energies and with a varying amount of filtered MOs. In the top row of Figure 7, we show the current density calculated at *E*_F_ with no MOs filtered. In the middle row of Figure 7, we show the current density calculated at the energy of the HOMO, *E*_HOMO_. In the bottom row of Figure 7, we show the current density calculated at *E*_HOMO_, where the contributing MOs have been filtered according to eq 25. Choosing *x* = 0.9 is equal to only including two orbital–gradient pairs in the current density calculation: ψ_HOMO_∇ψ_HOMO–1_ and ψ_HOMO–1_∇ψ_HOMO_.

**Figure 6:**
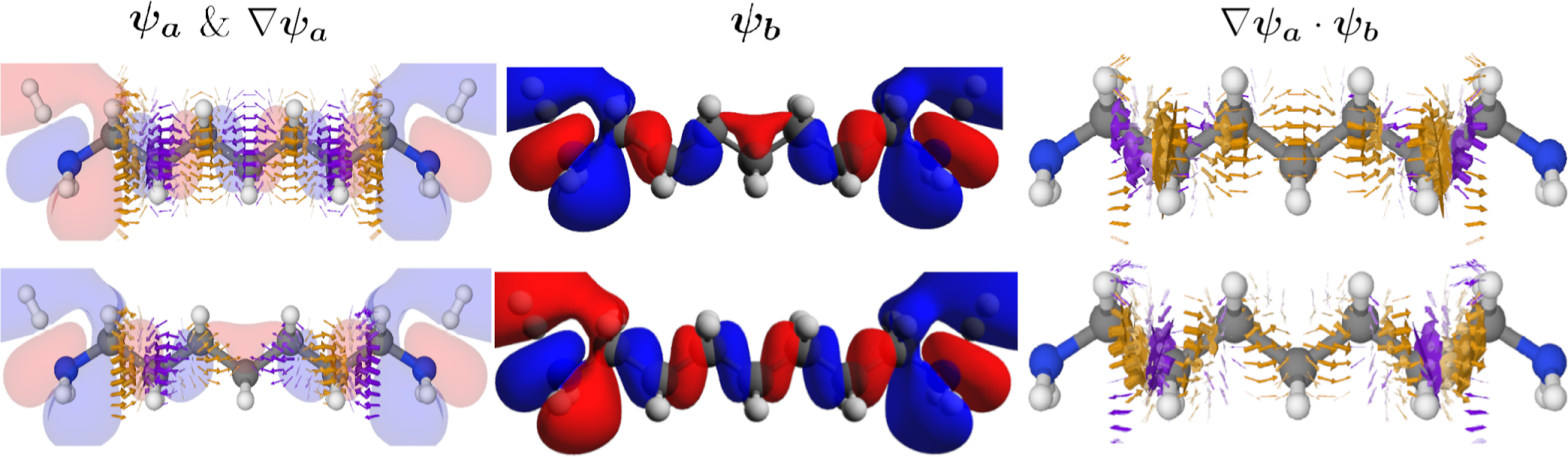
Two contributions to the current density calculated at *E*_HOMO_ for **2**. The top row depicts (left) the HOMO and its gradient as a vector field, (middle) HOMO – 1, and (right) the result of multiplying the HOMO – 1 and the gradient of the HOMO. The bottom row depicts (left) the HOMO – 1 and its gradient, (middle) HOMO, and (right) the result of multiplying the HOMO – 1 and the gradient of the HOMO. The bottom row of ∇ψ_*a*_·ψ_*b*_ has been multiplied by −1 to match how its contribution to the overall current density. Arrows smaller than 2.5% of the largest arrow have been omitted. The vector fields are colored by the normalized *z*-component. Isosurface: 0.01 au.

**Figure 7:**
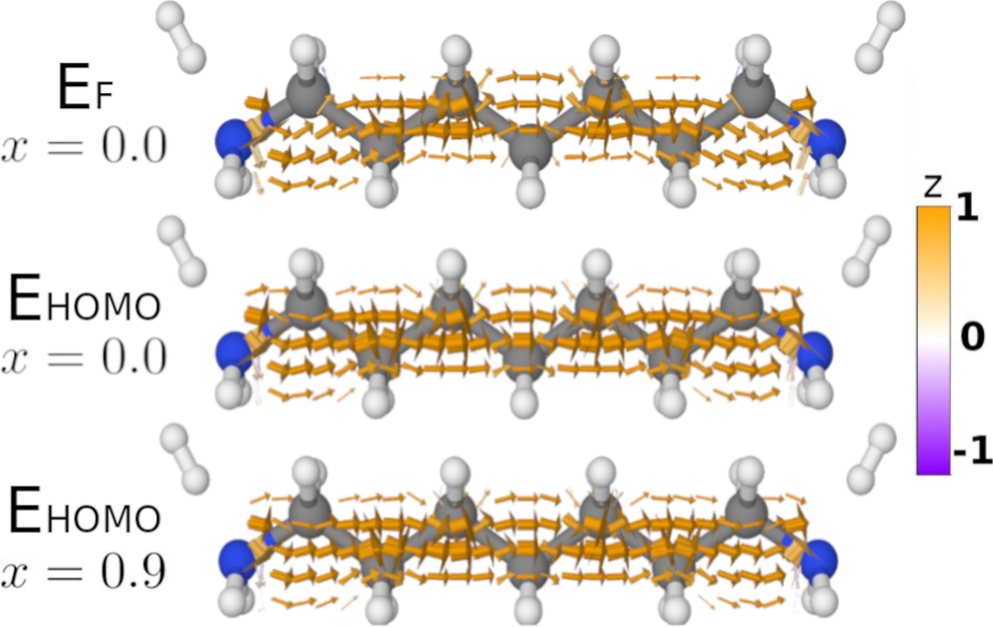
Illustration of the qualitative change in the current density of **2** when calculated at *E*_F_ with all MOs compared with the current density calculated at *E*_HOMO_ with only two MO-gradient pairs. The top row shows the current density at *E*_F_ with all MOs included; the middle row shows the current density at *E*_HOMO_ with all MOs included; and the bottom row shows the current density calculated at *E*_HOMO_ where **G**_C_^<^ has been filtered according to eq 25. Arrows smaller than 5% of the largest arrow have been omitted.

To understand what makes the current density curve away from the bonds, we start by reducing the number of orbital–gradient pairs included in the calculation. This reduction is done in two steps: First, we calculate the current density at the energy of the *E*_HOMO_. We see from the middle row of Figure 7 that this has a negligible effect on the qualitative shape of the current density. Second, we filter out all but two orbital–gradient pairs: ψ_HOMO_∇ψ_HOMO–1_ and ψ_HOMO–1_∇ψ_HOMO_. Again, there is a negligible effect on the qualitative shape of the current density.

In Figure S11, we show that this reduction is also quantitatively justified. The integrated current from the current density along the transport direction is, in effect, the same whether the current density at *E*_HOMO_ is calculated with all orbital–gradient pairs or with only the two selected orbital–gradient pairs.

In Figure 6, we plot the components of the two selected orbital–gradient pairs. In the left column, we plot ∇ψ_*a*_ as a yellow-purple vector field of the HOMO and the HOMO – 1 in the top and bottom rows, respectively. We have superimposed ψ_*a*_ on top of the vector field in transparent red and blue. Across the nodes, there are large gradients (large arrows) as we go from an area with a positive phase to a negative phase and vice versa.

In the middle column, we show the MO, ψ_*b*_, which makes up the other half of the MO-gradient pair: the HOMO – 1 in the top row and the HOMO in the bottom row. Contrary to the nodal plane in the plane of **1**, there are no nodes in the same position between the HOMO and the HOMO – 1.

The combination of the two orbital–gradient pairs is shown in the last column of Figure 6. The contribution from ∇ψ_HOMO_· ψ_HOMO–1_ is shown in the top, and ∇ψ_HOMO–1_ ·ψ_HOMO_ is shown in the bottom. Notably, ψ_*b*_ accentuates regions of the gradient with large isovalues (lobes) and limits contributions from regions with isovalues near or at zero (nodes). Although this is the same fundamental mechanism driving the current density of **1**, the result is different. For **1**, the non-overlapping nodal planes between bonds in its MOs (as visualized in the HOMO and HOMO – 1 in Figure 4) lead to a consequent absence of nodal planes between bonds in the resulting current density. In contrast, for **2**, the combined effect of the gradients across the nodal planes and the MOs results in the current density curving away from the bonds.

This interaction between the MOs and their gradients also explains why the current density flows away from the bonds even when we exchange all the carbons with silicon or germanium;^19^ their frontier orbitals do not qualitatively change, so the current density does not qualitatively change either.

It is clear that the current density still flows away from the bonds even when we only include ψ_HOMO_ and ψ_HOMO–1_ in the calculation of the current density. As such, it is not an emergent property stemming from the combination of multiple MOs but is an inherent consequence from specific orbital–gradient pairs.

### How Does Circular Current Emerge from Linear Systems?

In α, ω-disubstituted even [*n*]cumulenes, the frontier orbitals are helical with alternating chirality. If the HOMO is a P-helix, the HOMO – 1 is an M-helix, and the same principle applies to the virtual orbitals. Examples of helical MOs are shown in the right column of Figure 4 and the middle column of Figure 9, and their origin has been explained in detail earlier.^25^

**Figure 8:**
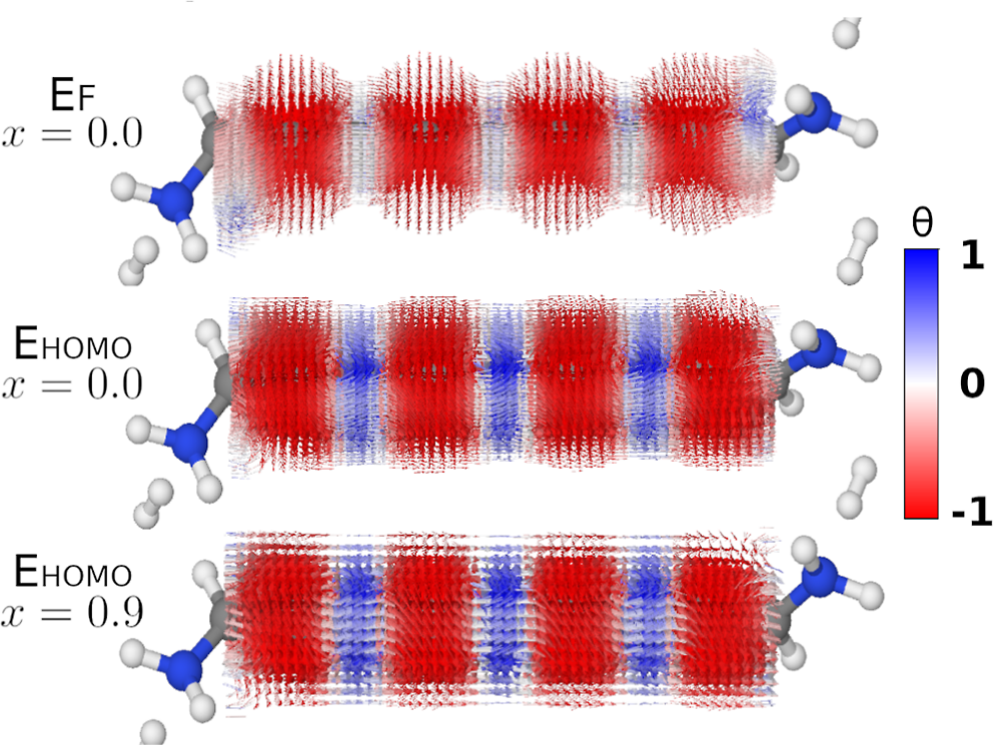
Illustration of the qualitative change in the current density of **3** when calculated at *E*_F_ with all MOs compared with the current density calculated at *E*_HOMO_ with only two MO-gradient pairs. The top row shows the current density at *E*_F_ with all MOs included; the middle row shows the current density at *E*_HOMO_ with all MOs included; and the bottom row shows the current density calculated at *E*_HOMO_ where **G**^<^′ has been filtered according to eq 25. Arrows smaller than 5% of the largest arrow have been omitted.

**Figure 9:**
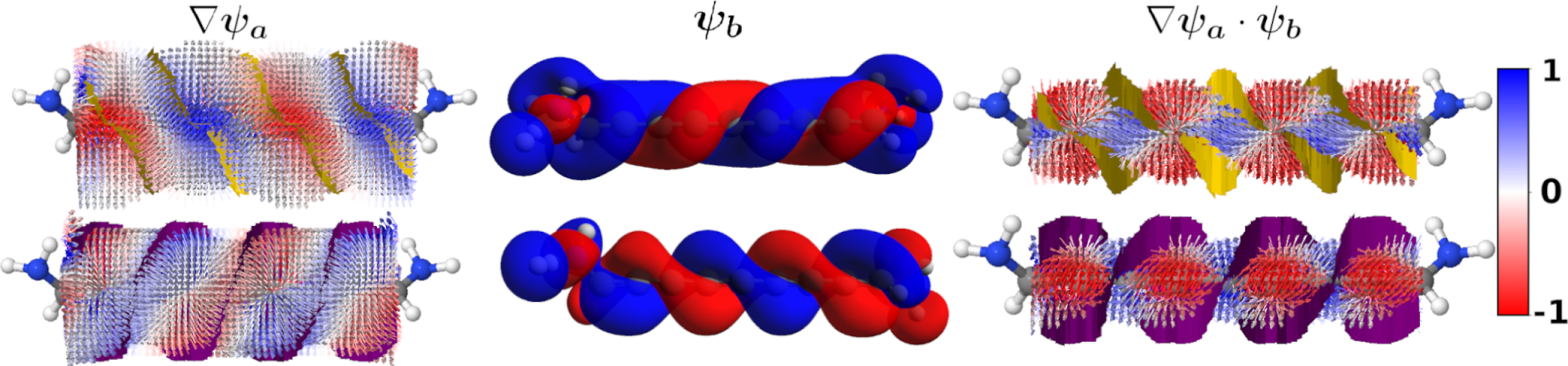
Two contributions to the current density calculated at *E*_HOMO_ for **3**. The top row depicts (left) the gradient of the HOMO (vector field) and its nodal plane (yellow plane), (middle) HOMO – 1, and (right) the result of multiplying the HOMO – 1 and the gradient of the HOMO. The bottom row depicts (left) the gradient of the HOMO – 1 and its nodal plane (purple plane), (middle) HOMO, and (right) the result of multiplying the HOMO and the gradient of the HOMO – 1. The bottom row of ∇ψ_*a*_·ψ_*b*_ has been multiplied by −1 to match its contribution to the overall current density. Arrows smaller than 10% of the largest arrow have been omitted. The vector fields are colored by the normalized θ-component. Isosurface: 0.01 au.

To highlight the circular component of the current density in **3**, we change the coloring of the arrows of the current density. Instead of a Cartesian coordinate system where coloring is by the *z*-component, we change to a cylindrical coordinate system where coloring is by the ϕ-component.

In the following, we will explain three effects seen in the current density of even [*n*]cumulenes using the MOs and their gradients: why the current density, at *E*_HOMO_, changes direction at every other atom; why no nodal plane is seen in the current density; and why circular current density emerges from helical MOs. To answer these questions, we simplify the analysis of the current density by calculating it at *E*_HOMO_ using only ψ_HOMO_ and ψ_HOMO–1_.

In Figure 8, we show how the current density of **3** changes when calculated in three different ways: at *E*_F_ with all MOs included (*x* = 0.0); at *E*_HOMO_ with all MOs (*x* = 0.0); and at *E*_HOMO_ with only the two HOMOs (*x* = 0.9). The biggest qualitative change happens when moving from *E*_F_ to *E*_HOMO_ as the circular current density starts alternating between moving clockwise and counterclockwise around every other carbon atom. This change is visualized by the interchanging red and blue arrows. It was also reported by Garner et al.^20^

When we reduce the description of the current density calculated at *E*_HOMO_ to only include the two HOMOs, we see that the vector field thins out. We show in Figure S12 that the integrated current along the *z*-direction is lower when we only use ψ_HOMO_ and ψ_HOMO–1_ to calculate the current density. Combined with the fact that we disregard arrows smaller than 5% of the largest arrow, fewer arrows are plotted. While the filtered current density deviates quantitatively from the full current density, the qualitative structure is preserved, as seen from Figure 8.

The alternating, circular current density is a result of ψ_*a*_ having one chirality and ψ_*b*_ having the opposite chirality. In the left column of Figure 9, labeled ∇ψ_*a*_, we see that the gradient of ψ_*a*_ has some notion of circularity (red and blue arrows) and that it follows the helically propagating nodal planes (the yellow plane in the top row and the purple plane in the bottom row). In the two previous examples, we already showed and explained why the gradient across a nodal plane is significant. Next, we look at the middle column, labeled ψ_*b*_, and note that the overall shape of the MO is a helix with opposite chirality compared with ψ_*a*_ of ∇ψ_*a*_. Lastly, the rightmost column shows the result when we multiply ∇ψ_*a*_ with ψ_*b*_. Focusing on the top row, we see that ψ_*b*_ emphasizes the region of space that the current density occupies, while the gradient dictates the flow of the current density in this region. Whether the current flows clockwise or counterclockwise around the carbon chain is determined by the signs of ∇ψ_*a*_ and ψ_*b*_. For example, a red area of ∇ψ_*a*_ multiplied by a red area in ψ_*b*_ will give a blue area in ∇ψ_*a*_·ψ_*b*_. The alternating, circular flow of the current density is inherently tied to the orbital–gradient pairs of helical MOs, though the degree of alternation might depend on the degree of helicality of the MOs.^62^

Next, we explain why we do not see the nodal plane in the resulting current density. It is clear from both Figures 8 and 4 that there is no nodal plane in the current density. The only two contributions to the approximated current density are the ones shown in the last column of Figure 9. If we add these together, we get exactly the current density that is shown at the bottom of Figure 8. The main qualitative difference between the two contributions is the spatial distribution of the current density; where one has a low density, the other has a large. Alternatively, we can look at just the ψ_*b*_s, as these emphasize the path that the current density follows. The maximum of one MO coincides with the nodal plane of the other. Wherever the first MO eliminates the current density from its contribution, the other MO emphasizes the current density in exactly that location. This oscillating pattern of emphasis arises due to the differing helical chiralities of the two MOs by symmetry.

The last observed phenomenon, the emergence of circular current from helical MOs, is most clearly seen in Figure 8 and the two contributions of the rightmost column of Figure 9. To explain this phenomenon, we start by looking at ∇ψ_*a*_ in the left column of Figure 9. As we explained earlier, the circular component of the gradient field is already notable, as seen by the blue and red arrows that cluster along the nodal planes. We also explained that the MO, ψ_*b*_, emphasizes the region of space that the circular current density occupies. Importantly, because ψ_*b*_ is guaranteed by symmetry to have opposite chirality compared with ψ_*a*_, ψ_*b*_ will not suppress all the regions where ∇ψ_*a*_ has a high density of arrows. The result of this can be seen in the rightmost column of Figure 9. Ultimately, the circular current arises from the gradient across a nodal plane but is not inherently tied to the helicality of the MO. Although the circular current manifests from the gradient across a nodal plane, it is not intrinsically linked to the helical nature of the MO. The helical MO serves to facilitate the propagation of the circular current from the gradient field to the current density. However, it is not directly responsible for the circular current density itself. Rather, the combined action of the two MOs, their respective chiralities, and their interaction with nodal planes and gradients contribute to this unique phenomenon of circular current density.

## Conclusions

In this paper, we have shown that the current density can be understood from an orbital point of view as long as both the orbital and its gradient are considered equally. Furthermore, as only off-diagonal orbital–gradient pairs contribute to the current density, this gives rise to the counter-intuitive current density seen in alkanes **2** and even [*n*]cumulenes **3,** while still conforming to our expectation in alkenes **1**.

For the alkene system **1**, the nodes in the plane of the molecule show up in the current density, while the inter-lobal nodes do not. We have shown that it is possible to calculate the current density of the σ- and π-system separately. This separation meant that we were able to only focus on the π-system almost without approximation of the current density. By focusing on the π-system, we were able to explain the nodal plane in the current density by the fact that all π-MOs have their nodal plane in the same place.

For the alkane system **2**, the current density could be explained by considering the relative position of the nodes in each of the orbital–gradient pairs. As an example, we calculated the current density at the energy of the HOMO and saw that the description of the current density could be reduced to that of the HOMO and the HOMO – 1. As these two MOs have nodes in different places, the contribution from the gradient across the nodes will be accentuated.

In the even [*n*]cumulene **3**, we explained three effects: why the circular current density of even [*n*]cumulenes, when calculated at *E*_HOMO_, changes direction at every other atom; why we do not see the helical nodal plane in the current density; and why the current density flows circularly when the MOs are helical.

The alternating, circular current density is directly tied to each MO-gradient pair of helical MOs and not from a combination of many MOs. It happens due, in part, to the HOMO and HOMO – 1 having opposite helical chiralities. The alternating, circular flow of the current density is tied to the helical MOs, but it might be that the degree of alternation depends on the degree of helicality of the MOs.

To explain why we do not see the nodal plane in the current density, we made use of the same argument as for why we do not see the inter-lobal nodal planes in **2**. As the helically propagating nodal planes of HOMO and HOMO – 1 are, by symmetry, guaranteed to be mirror images of each other, ψ_*b*_ will primarily emphasize the current density, where the other will have suppressed almost everything and vice-versa. Thus, the contribution to the overall current density from one MO-gradient pair has its maximum where the other MO-gradient pair has its minimum.

The last effect of circular current arising from helical MOs was explained by realizing that the flow of the current density arises from the gradient across a nodal plane. The circularity is not tied directly to the helicality of the MO, but as ψ_*a*_ and ψ_*b*_ will have opposite chirality by symmetry, the gradient across the nodal plane of ψ_*a*_ will not be suppressed by the nodal plane of ψ_*b*_, as we saw in **2**.

From eq 24, ψ can be thought of as a scalar field, while the gradient of such a scalar field is a vector field. Therefore, ψ provides static information, and the gradient of ψ provides dynamic information about the system. Despite the success of MOs to explain steady-state systems, inserting a molecule between two electrodes and applying a bias across that molecule is a dynamical system, and as such, we need the dynamical information to fully describe the effects we see.

While this role of nodal planes in MOs may be surprising in the realm of current density, the significance of nodal planes is not a novelty in chemical theory. They act as delineations within MOs, carving out regions of electron probability density, and thus are instrumental in explaining a multitude of fundamental chemical phenomena such as bonding and spectroscopic properties. Our study does not merely elucidate the behavior of current density in dynamical systems; instead, it provides a connection between current density and the age-old concepts of MOs and nodal planes. Consequently, this work bolsters the foundational understanding of chemical theory, uniting static and dynamic phenomena under a simple principle. By using MOs as a direct explanation for current density, we hope to narrow the gap between chemists and physicists, enabling a more diverse approach to single-molecule electronic devices.

## Supporting Information Available

Rotation of basis, visualizing the nodal plane, coordinate transformation, octane, current density convergence, polarization functions in octatetraene, and current density after filtering (PDF)
