# Supplementary material for: Understanding Current Density in Molecules Using Molecular Orbitals
Source: J Phys Chem A. 2023 Oct 19;127(43):9003–12. doi: 10.1021/acs.jpca.3c04631 (PMC10627148; doi:10.1021/acs.jpca.3c04631)
Supplement: Supplementary file 1 — jp3c04631_si_001.pdf [file jp3c04631_si_001.pdf]

Supporting Information: Understanding Current Density In Molecules Using Molecular Orbitals

William Bro-Jørgensen and Gemma C. Solomon*

Department of Chemistry and Nano-Science Center, University of Copenhagen, Universitetsparken 5, DK-2100, Copenhagen Ø, Denmark

E-mail: gsolomon@chem.ku.dk

Rotation of basis

A general formula for rotating from one basis to another is given in Equation 1

$$\mathbf{A} = \mathbf{v}^{\dagger}\mathbf{A}\mathbf{v} \tag{1}$$

where $\mathbf{v}$ is the eigenvectors of the new basis and $\mathbf{A}$ is a matrix that needs to be rotated from one basis to another. To get the necessary eigenvectors, the following equation can be used:

$$\mathbf{S_{AO}}^{-1}\mathbf{H_{AO}} = \epsilon\mathbf{v} \tag{2}$$

Here, S_{AO} is the overlap matrix in an atomic orbital basis (AO), H_{AO} is the Hamiltonian in an AO basis, ϵ is the molecular orbital (MO) energies and $\mathbf{v}$ is the eigvenvectors used to rotate from an AO to an MO basis.

To calculate the current density in an MO basis, it is then necessary to rotate Γ_L, Γ_R,

S_{AO} and H_{AO}. After that, the current is calculated by the same procedure outlined in the manuscript.

Visualizing the Nodal Plane

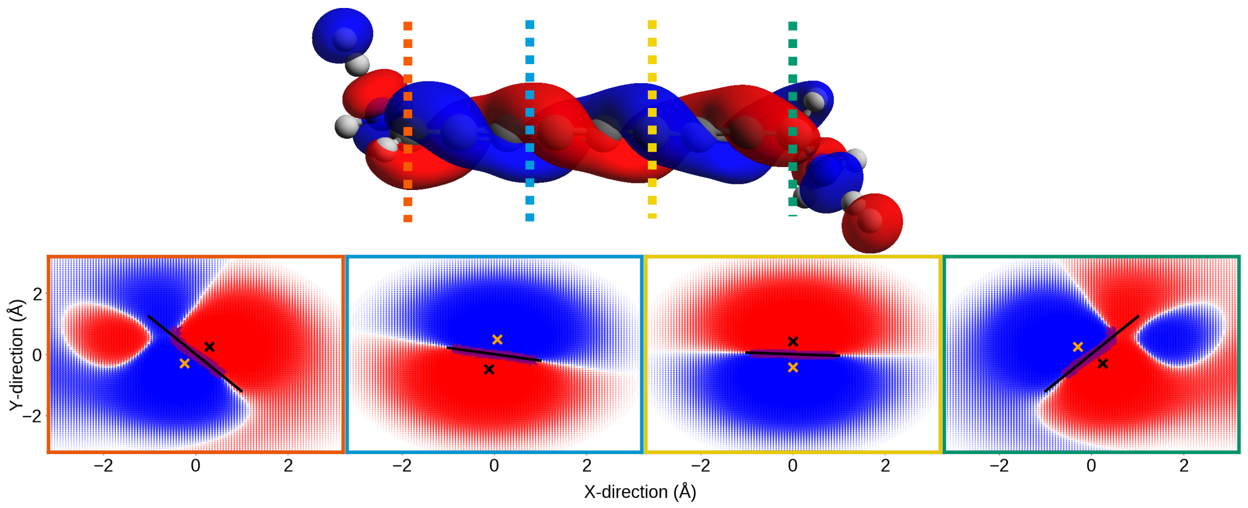

Figure S1: Four different xy-slices of the HOMO of **3**. The purple crosses denotes the isovalue crossing zero, the black line is a fitted straight line through the purple crosses and the orange and black cross denotes the highest and lowest isovalue in a short radius around the point (0, 0).

To find the nodal plane of a given MO requires a .cube file of that particular MO. These can be created by most quantum chemical programs, for example, by Gaussian using the `cubegen` tool. In Figure S1, the MO has been dumped using GPAW. The code to find the nodal plane of an arbitrary MO has been uploaded to Github alongside a short description of usage. It is available at `https://github.com/chem-william/find_nodal`.

First, the MO is sliced into xy-planes along the z-axis which in this case is chosen to be the carbon chain. Then, a subset of that slice is generated by filtering out any point that lies more than 0.9Å away from (0, 0). This distance is somewhat arbitrarily chosen as it only serves to make the following calculations of the nodal plane easier. A shorter or a longer distance could have been chosen with negligible change in the results. After filtering, every

sign change is located, i.e., the line at which the phase of the MO change. This gives two lists of points: one with points before a zero (list A) and one with points after a zero (list B). A new set of points are then calculated (list C) as the midway point between each set of points in list A and B. List C is the purple points shown in Figure S1. The points of list C are then fitted to a straight line parameterized according to Equation 3. This is the black line seen in Figure S1.

$$ax = y \tag{3}$$

The black line is now our new representation of the nodal plane in that slice which we can use to visualize the nodal plane of a given MO.

Coordinate Transformation

A conversion from Cartesian to cylindrical coordinates take the (x, y)-points and convert them to a radius, r, and an angle, ϕ. The polar (cylindrical) vector components can be calculated as

$$\vec{v}_{r,\phi} = (v_x \cos(\phi) + v_y \sin(\phi))\, \hat{e}_r + (v_y \cos(\phi) - v_x \sin(\phi))\, \hat{e}_\phi \tag{4}$$

where $\hat{e}_r$ and $\hat{e}_\phi$ are the cylindrical unit vectors, and v_x and v_y are the cartesian vector components. The carbon axis is aligned with the z-axis thus placing it in the origin of the cylindrical coordinate system.

Octane

We show in Figure S2 the two biggest contributions to the current density at E_{HOMO} for 1,8-diaminooctane. Compared with **2**, 1,8-diaminooctane does not have a nodal plane on the middle carbon atom. Instead, it has a nodal plane across the middle bond. Nevertheless, the overall picture of the current density is the same: it has a large gradient across the nodal

planes (see left column of Figure S2) and ψ_b seems to pick out the regions of space that the current density occupies (see right column of Figure S2).

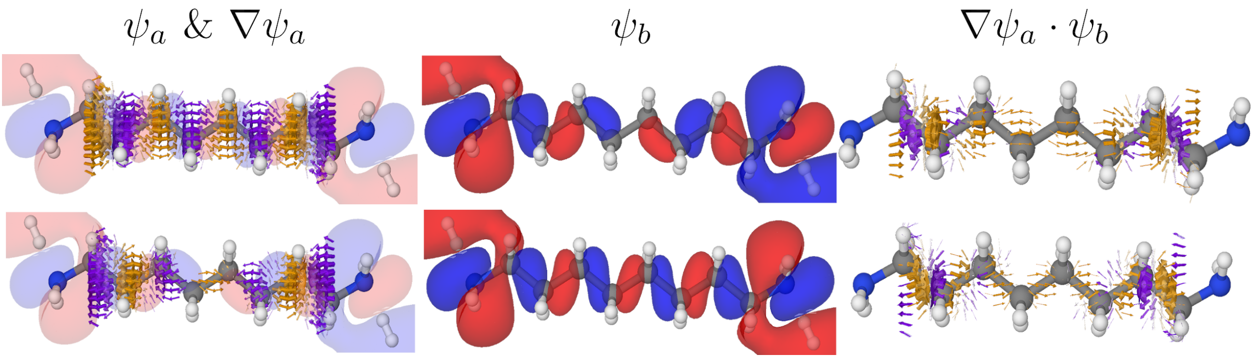

Figure S2: The two contributions to the current density calculated at E_{HOMO} for 1,8-diaminooctane. The top row depicts (left) the HOMO and its gradient as a vector field, (middle) HOMO − 1 and (right) the result of multiplying the HOMO − 1 and the gradient of the HOMO. The bottom row depicts (left) the HOMO − 1 and its gradient, (middle) HOMO and (right) the result of multiplying the HOMO − 1 and the gradient of the HOMO. The vector fields are colored by the normalized z-component. Isosurface: 0.02 au.

Current Density Convergence

It has been described in recent work that the current density may not preserve the total current throughout the molecule.[S1,S2] It has been suggested that it is because of the finite local basis set though no conclusive answer have been put forth. The divergence of the current density can be estimated by integrating the current density, $j(\mathbf{r})$ over a plane, $\mathbf{A}$, perpendicular to the transport direction z.

$$J = \int d\mathbf{A} j(\mathbf{r}), \quad d\mathbf{A} = dxdy \tag{5}$$

The current through the plane, J, can be compared with the total current, I, which can be calculated using the general Landauer-Büttiker formula

$$I = \frac{e\hbar}{2\pi} \int dE \left(f_L(E) - f_R(E)\right) T(E) \tag{6}$$

where T(E) is the transmission function and $f_{L/R}$ are the Fermi functions of the left and right electrodes.

The two methods are physically equivalent, i.e., $J = I$, but when J is calculated for xy-planes along the z-axis, it can be seen that J diverges when compared to I. This is shown in Figures S3 to S8

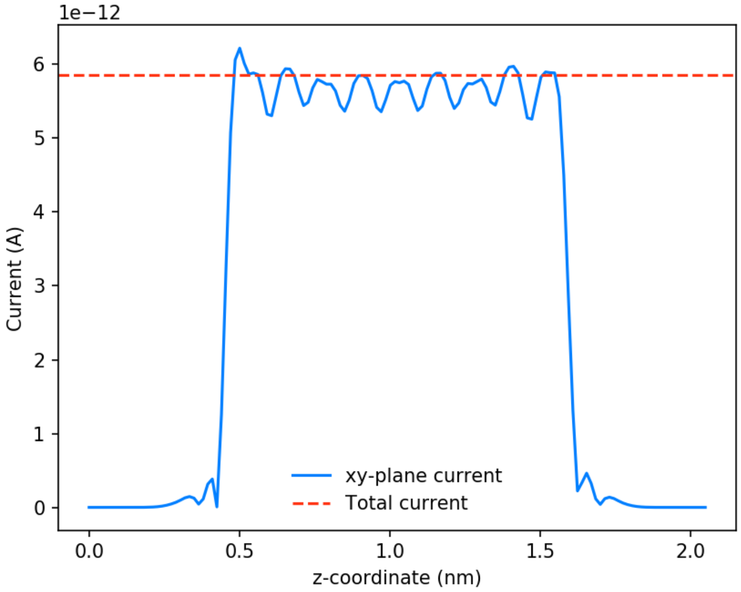

Figure S3: Total current and integrated current from the current density as a function of the z-coordinate (transport direction) calculated for 1,8-diaminooctatetraene (**1**) at the Fermi energy.

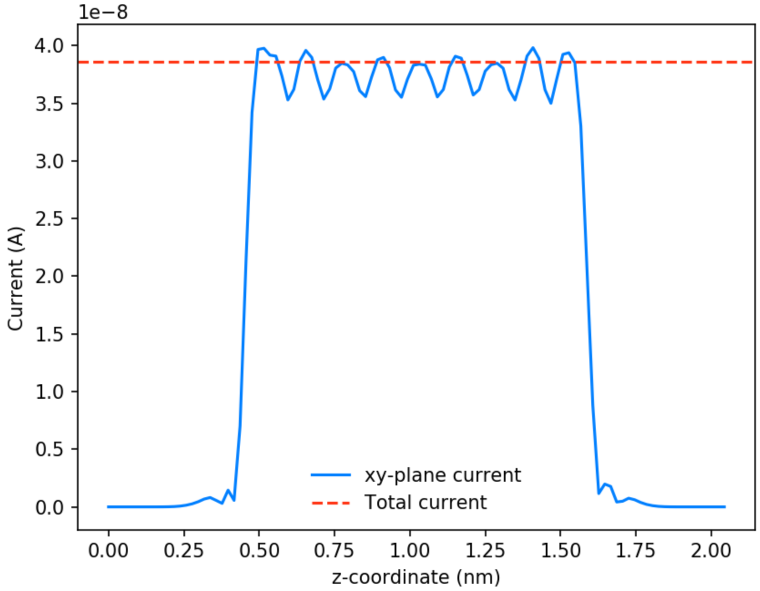

Figure S4: Total current and integrated current from the current density as a function of the z-coordinate (transport direction) calculated for 1,8-diaminooctatetraene (**1**) at the energy of the HOMO.

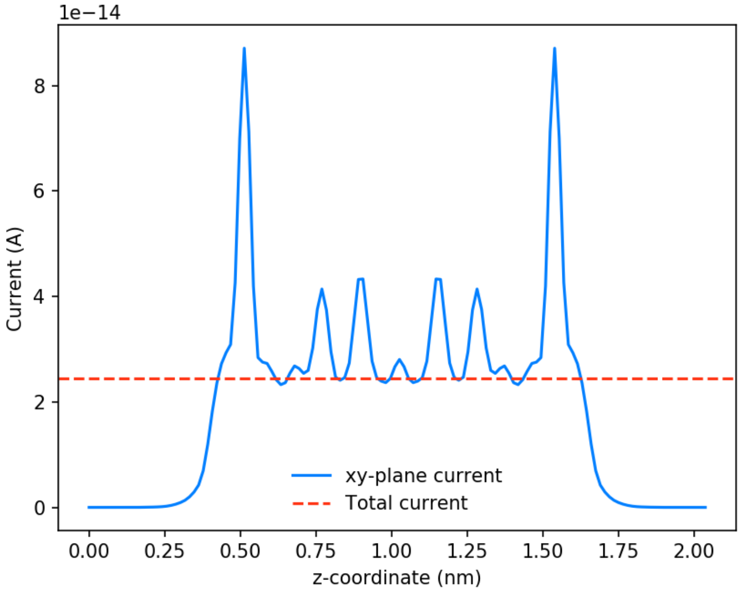

Figure S5: Total current and integrated current from the current density as a function of the z-coordinate (transport direction) calculated for hept-1,7-diamine (**2**) at the Fermi energy.

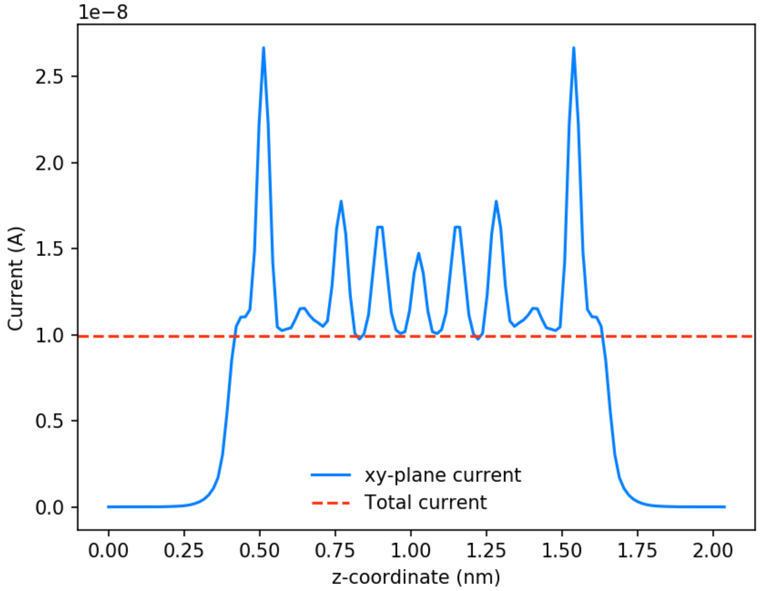

Figure S6: Total current and integrated current from the current density as a function of the z-coordinate (transport direction) calculated for hept-1,7-diamine (**2**) at the energy of the HOMO.

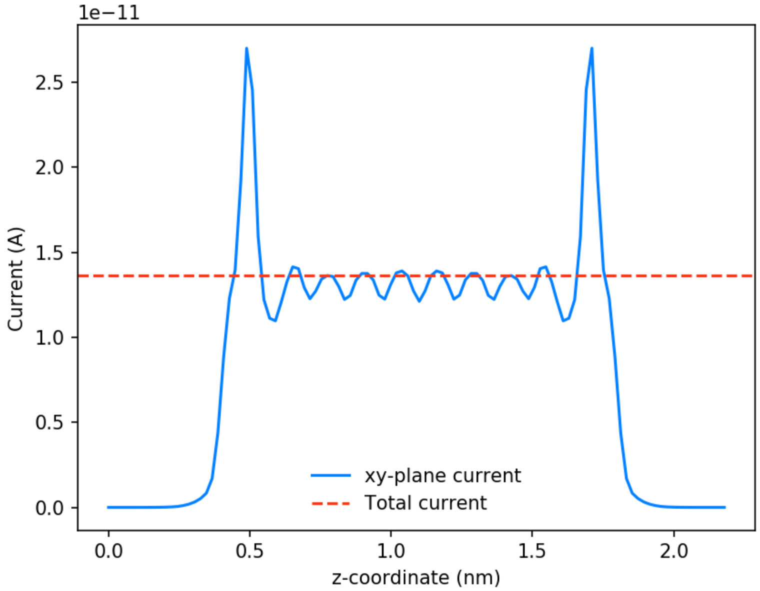

Figure S7: Total current and integrated current from the current density as a function of the z-coordinate (transport direction) calculated for R-1,9-diamino-[8]cumulene (**3**) at the Fermi energy.

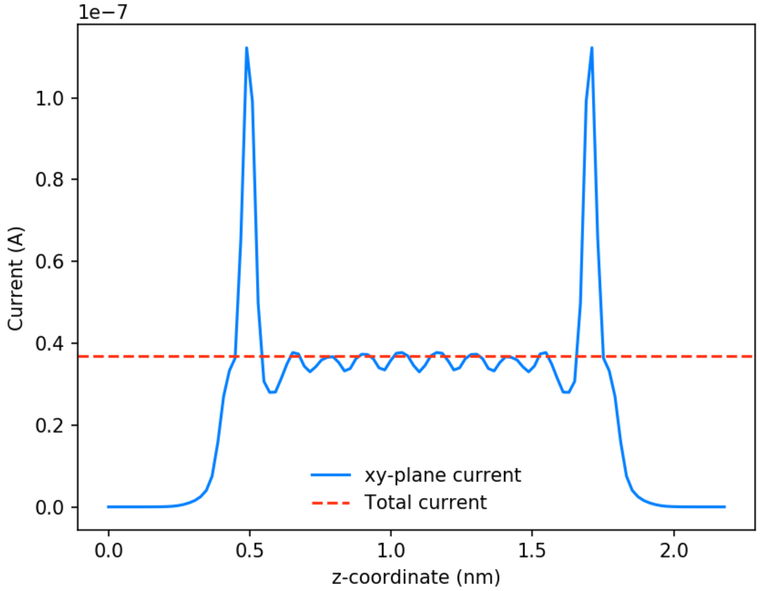

Figure S8: Total current and integrated current from the current density as a function of the z-coordinate (transport direction) calculated for R-1,9-diamino-[8]cumulene (**3**) at the energy of the HOMO.

Polarization functions in octatetraene

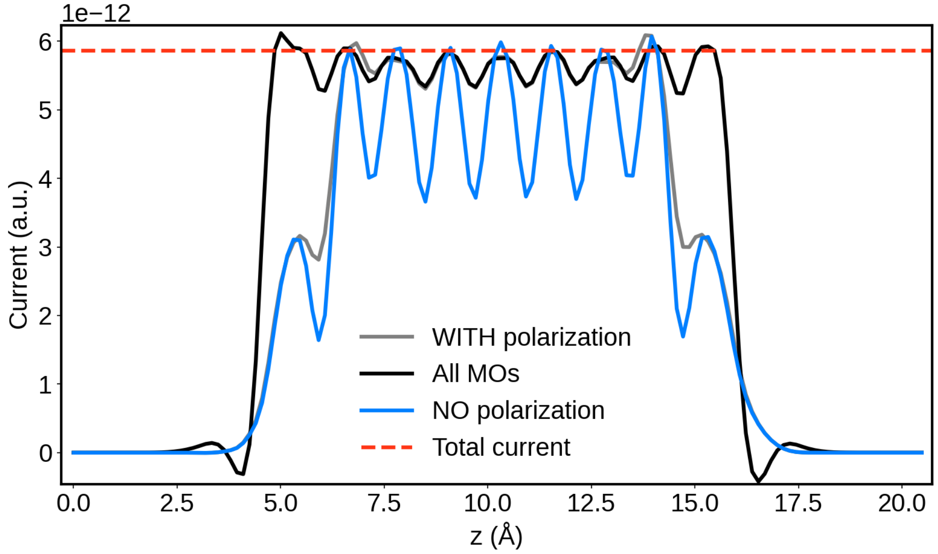

Figure S9: Total current (red, dashed line) and integrated current from the current density calculated with different numbers of MOs included: all MOs (black, solid line); only π-system where polarization functions have been included (gray, solid line); and π-system where polarization functions have been omitted. Calculated for 1,8-diaminooctatetraene (**1**) at the Fermi energy.

In Figure S9, we show the integrated current from the current density calculated with different numbers of MOs included in the calculation: all MOs (black, solid line); only π-system where polarization functions have been included (gray, solid line); and π-system where polarization functions have been omitted. We can see that going from the full MO-picture to only considering the π-system mostly affects the current at the electrodes (around 5.0 and 15.0 Å on the x-axis of Figure S9). When we omit the polarization functions from the current density, we see that the integrated current deviates considerably from the all-MO integrated current and as such, a notable amount of current through the π-system is explained by the polarization functions.

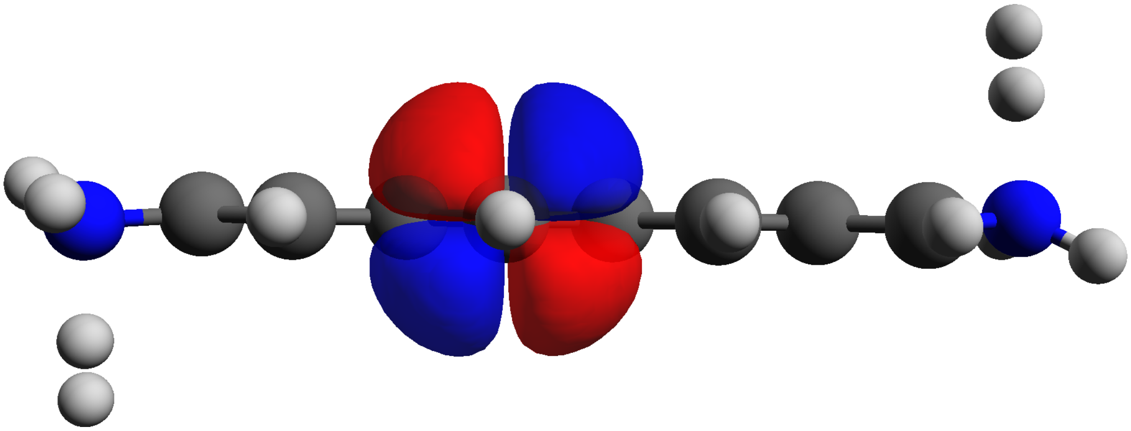

Figure S10: Plot of d_{zz}-orbital. Isosurface: 0.02 au.

In Figure S10, we show a d_{zz}-orbital. As we explain in the main text, this AO has a nodal plane in the molecular plane. This is the same plane where a p_z-orbital has its nodal plane thus the conclustion from the main text, that all MOs of the π-system has a nodal plane in the same spot, remains the same.

Current Density after Filtering

In Figure S11, we show the total current calculated with all MOs included and the integrated current from the current density where we have varied the value of x meaning that we filter out more and more MOs, the higher the value of x becomes. As can be seen from the turquoise and black line, they almost overlap throughout the molecule, suggesting that the current density at E_{HOMO} can be described by only the two biggest contributions almost with no approximation.

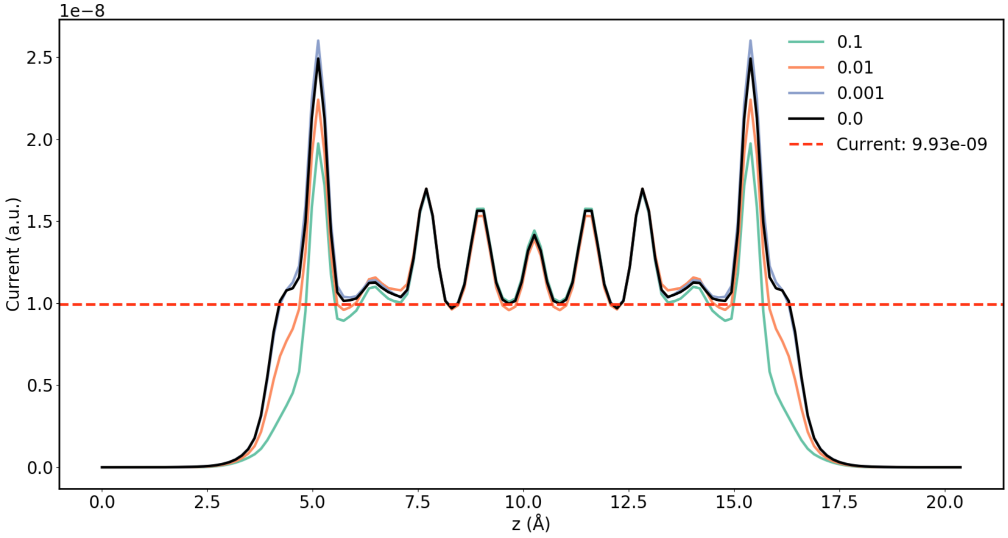

Figure S11: Total current calculated with all MOs and integrated current from the current density calculated at different values of $1 - x$. Both calculated as a function of the z-coordinate (transport direction) for hept-1,7-diamine (**2**) at E_{HOMO}.

By filtering according to Equation 25 in the main manuscript, we also have information about the exact molecular orbitals that have been included in the current density calculation. Even though this information will be subject to change if we use another basis set when doing the calculations, we feel it is instructive to include here.

For **2**, where the current density have been calculated at E_{HOMO} and $x = 0.9$, these are shown in Table S1. We see that the only MOs that are included in the calculation is 29 and 28 which are the HOMO and HOMO − 1 respectively.

Table S1: Table of MO-gradient pairs that contribute to the current density of hept-1,7-diamine (**2**) at E_{HOMO} when $x = 0.9$

MO-pairs	
[28 29]	[29 28]
**Unique orbitals**	28, 29

If we decrease x to 0.09, we see in Table S2 that only two extra MO-gradient pairs contribute coming from a single, new MO. This MO, 58, is the LUMO + 28.

As we did for **2**, we show, in Figure S12, the total current calculated with all MOs included and the integrated current from the current density where we have varied the value of x.

Table S2: Table of MO-gradient pairs that contribute to the current density of hept-1,7-diamine (**2**) at E_{HOMO} when $x = 0.09$

MO-pairs				
	[28 29]	[29 28]	[29 58]	[58 29]
**Unique orbitals**	28, 29, 58			

This time we see that the turquoise and black line differs significantly from one another. As we argue in the main manuscript, this is not necessarily a problem as the qualitative characteristics is still present.

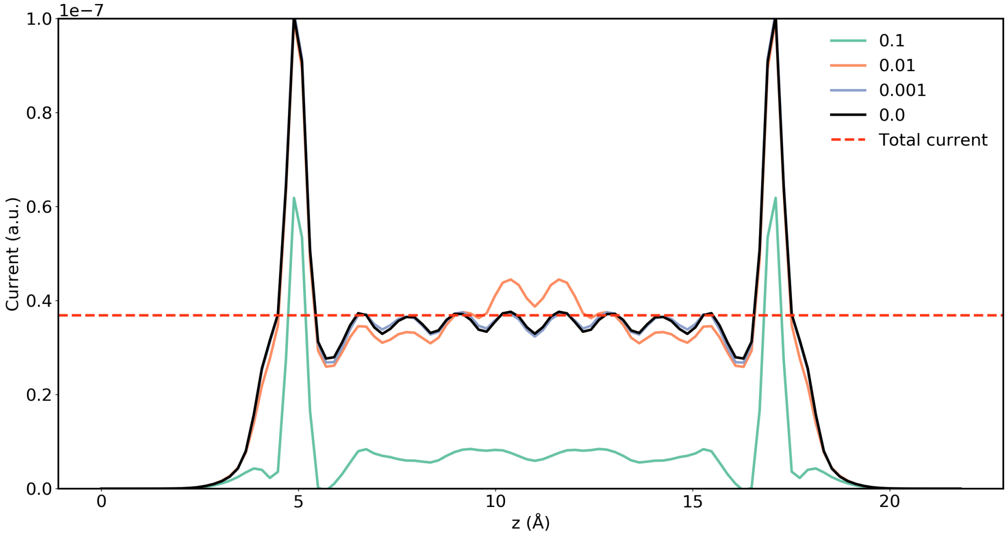

Figure S12: Total current calculated with all MOs and integrated current from the current density calculated at different values of $1 - x$. Both calculated as a function of the z-coordinate (transport direction) calculated for R-1,9-diamino-[8]cumulene at E_{HOMO}.

Table S3: Table of MO pairs that contribute to the current density of R-1,9-diamino-[8]cumulene (**3**) at E_{HOMO} when $x = 0.9$

MO-pairs	
[26 27]	[27 26]
**Unique orbitals**	26, 27

Here, MO 27 is the HOMO and 26 is the HOMO − 1.

Contrary to the case for **2**, where going from $x = 0.9$ to $x = 0.09$ only introduced one more MO, for **3**, it considerably increases the number of MOs that contribute to the current density as can be seen from Table S4

Table S4: Table of MO pairs that contribute to the current density of R-1,9-diamino-[8]cumulene (**3**) at E_{HOMO} when $x = 0.09$

MO-pairs			
[14 27]	[18 27]	[21 27]	[22 27]
[25 27]	[26 27]	[27 14]	[27 18]
[27 21]	[27 22]	[27 25]	[27 26]
[27 29]	[27 44]	[27 46]	[29 27]
[44 27]	[46 27]		
**Unique orbitals**	14, 18, 21, 22, 25, 26, 27, 29, 44, 46		

Again, MO 27 is the HOMO, MO 26 is the HOMO – 1, 25 is HOMO – 2 and so on in descending order. MO 29 is LUMO + 1, MO 44 is LUMO + 16 and MO 46 is LUMO + 18.

References

(S1) Lai, L. Q.; Chen, J.; Liu, Q. H.; Yu, Y. B. Charge nonconservation of molecular devices in the presence of a nonlocal potential. *Physical Review B* **2019**, *100*, 1–7.

(S2) Jensen, A.; Garner, M. H.; Solomon, G. C. When Current Does Not Follow Bonds: Current Density in Saturated Molecules. *The Journal of Physical Chemistry C* **2019**, *123*, 12042–12051.
